# MRSA prevalence in european healthcare settings: a review

**DOI:** 10.1186/1471-2334-11-138

**Published:** 2011-05-20

**Authors:** Madeleine Dulon, Frank Haamann, Claudia Peters, Anja Schablon, Albert Nienhaus

**Affiliations:** 1 Institution for Statutory Accident Insurance and Prevention in the Health and Welfare Services. Department of Occupational Health Research, Pappelallee 33/35/37, 22089 Hamburg, Germany; 2 University Medical Center Hamburg-Eppendorf, Institute for Health Services Research in Dermatology and Nursing, Martinistr. 52, 20246 Hamburg, Germany

## Abstract

**Background:**

During the past two decades, methicillin-resistant *Staphylococcus aureus *(MRSA) has become increasingly common as a source of nosocomial infections. Most studies of MRSA surveillance were performed during outbreaks, so that results are not applicable to settings in which MRSA is endemic. This paper gives an overview of MRSA prevalence in hospitals and other healthcare institutions in non-outbreak situations in Western Europe.

**Methods:**

A keyword search was conducted in the Medline database (2000 through June 2010). Titles and abstracts were screened to identify studies on MRSA prevalence in patients in non-outbreak situations in European healthcare facilities. Each study was assessed using seven quality criteria (outcome definition, time unit, target population, participants, observer bias, screening procedure, swabbing sites) and categorized as 'good', 'fair', or 'poor'.

**Results:**

31 observational studies were included in the review. Four of the studies were of good quality. Surveillance screening of MRSA was performed in long-term care (11 studies) and acute care (20 studies). Prevalence rates varied over a wide range, from less than 1% to greater than 20%. Prevalence in the acute care and long-term care settings was comparable. The prevalence of MRSA was expressed in various ways - the percentage of MRSA among patients (range between 1% and 24%), the percentage of MRSA among *S. aureus *isolates (range between 5% and 54%), and as the prevalence density (range between 0.4 and 4 MRSA cases per 1,000 patient days). The screening policy differed with respect to time points (on admission or during hospital stay), selection criteria (all admissions or patients at high risk for MRSA) and anatomical sampling sites.

**Conclusions:**

This review underlines the methodological differences between studies of MRSA surveillance. For comparisons between different healthcare settings, surveillance methods and outcome calculations should be standardized.

## Background

*Staphylococcus aureus *(*S. aureus*) is a versatile human pathogen that causes diseases ranging from relatively mild infections of the skin and soft tissue to life-threatening sepsis [1]. The emergence of strains resistant to methicillin and other antimicrobial agents has become a major concern, especially in the hospital environment, because of the higher mortality due to systemic methicillin-resistant *Staphylococcus aureus *(MRSA) infections [2]. Tiemersma et al. [3] have shown significant increases in methicillin resistance in clinical strains of *S. aureus *isolates between 1999 and 2002 in European countries, particularly Belgium, Germany, Ireland, the Netherlands and the United Kingdom. MRSA prevalence varied widely, from < 1% in northern Europe to > 40% in southern and western Europe [3]. As the prevalence of healthcare-associated infections (HAIs) caused by multidrug-resistant organisms continues to increase [4], it seems essential to prevent MRSA transmission and reduce the number of MRSA HAIs. It is also important for healthcare workers that MRSA rates should be controlled, as a recently published review has shown that the average MRSA carrier rate in healthcare workers is 4.6%, and that about 5.1% of these carriers had symptomatic MRSA infections [5]. Although most MRSA infections in healthcare workers had a mild clinical course, some infections tend to become chronic and can cause severe health problems. This may lead to long-term incapacity, as has been shown by an analysis of the database of a German workers' compensation board [6].

For healthcare facilities, surveillance is an important and generally accepted method to assess the incidence of infection due to multidrug-resistant bacteria and - if necessary - to improve infection control measures [7]. Surveillance of MRSA is a means of identifying colonized or infected patients for whom specific control measures may be implemented [8]. Surveillance may be passive, whereby laboratory results from clinical samples are monitored, or active, whereby patients are actively screened for the carrier state in order to identify the entire reservoir. The implementation of a program of active surveillance cultures beside contact precautions is recommended by different national guidelines as a way of preventing nosocomial transmission of MRSA [9-12]. However, it is difficult to determine the range of MRSA rates from existing literature [13], as surveillance is primarily performed during outbreaks and generalization of these results is hampered, as the findings are not applicable to non-outbreak situations [8]. Moreover, as the recognition of MRSA as a hospital problem largely depends on clinical samples or swabs taken either only on admission or selectively for high risk patients, the true case load of a hospital or a specific setting remains largely unknown [14].

In the absence of comprehensive studies on the prevalence of MRSA, rates from MRSA surveillance in hospitals have been transferred to other healthcare settings. However, it has been questioned whether control measures in one setting can be generalized to other settings [9]. This question applies to both epidemic and endemic MRSA and also to specific settings, such as intensive care and non-acute wards, where MRSA may have widely variable transmission dynamics [8].

The aim of this review was to analyze the literature on MRSA prevalence in endemic (non-outbreak) situations in various clinical and long-term care (LTC) settings in European countries. Special emphasis was given to the calculation of MRSA outcomes and to MRSA screening methods.

## Methods

The search was conducted in the PubMed Medline database (2000 through June 2010) and was limited to articles in English or German. Inclusion and exclusion criteria were established on the basis of an exploratory search of the literature.

The search strategy used the keyword 'MRSA' combined with a group of screening-related terms: 'surveillance' OR 'infection control' OR 'prevalence' AND 'healthcare'. Primary inclusion criteria were developed for the initial selection of relevant articles, which were studies presenting MRSA prevalence rates. The initial search yielded 271 citations with an abstract. One of the authors (MD) screened the titles and the abstracts, in order to identify those that met the inclusion criteria, namely the prevalence of colonization or infection with MRSA in patients or residents in clinical and nursing healthcare settings. The exclusion criteria, with the number of excluded abstracts, were as follows: community-associated (n = 64), outbreaks (n = 41), study region outside Europe (n = 21), and healthcare personnel as the sole study population (n = 6). Another 128 abstracts were excluded as they failed to focus on MRSA prevalence. These included studies evaluating control measures, and those assessing the efficacy of special screening tests, or estimating MRSA transmission. The search was repeated in combination with a group of setting-related terms, namely 'long-term care' OR 'nursing home*' OR 'home care' OR 'mentally handicapped people', resulting in 65 additional abstracts of which 6 were included. Finally, the reference lists of the retrieved articles were checked for additional relevant studies, of which 15 were included. A total of 31 studies were included in the analysis.

Studies were separated into clinical and LTC settings. A data extraction form was developed to collect information from each selected study. This included the following points: 1) Study design: study period, study setting, and study population described by number of eligible participants and actual number of participants; 2) Screening policy: screening procedure and sampling sites; 3) Results: presented in the form of proportions (expressed as the percentage of MRSA cases among patients or among isolates of *S. aureus*) and density rates (expressed as MRSA cases per 1,000 patient days). The extraction of the data was performed by two authors (MD, FH).

The quality of each of the included studies was assessed by using seven criteria, taken from various checklists aimed either at evaluating prevalence surveys [15] or at improving the reporting of observational prevalence studies [16-19]. The criteria were expressed as questions (Table 1). For each study, the answers to these questions were graded as *Yes *if the question was satisfactorily answered (with a score of one point), *No *if it was not, and *'?' *for unclear answers or missing information (with a score of zero points). Two authors (CP, AS) independently assessed the study quality and a third (MD) resolved discrepancies. Study quality was categorized as good (> 5 points), fair (3 to 5 points) or poor (< 3 points). The criteria for rating the study quality in MRSA prevalence studies and the justification for a favorable evaluation will be described briefly.

**Table 1:** Checklist of criteria for assessing the quality of MRSA prevalence surveys

Number	Name	Content
1	Outcome definition	Was a valid definition given of the outcome for prevalence, colonization and infection?
2	Time unit	Was the endpoint calculated for a standardized time unit (daily, monthly, yearly)?
3	Target population	Was the target population specified by inclusion or eligibility criteria?
4	Participants	Was the number of included cases reported, e.g. by describing the numbers and reasons for non-participation?
5	Observer bias	Were sources of potential imprecision reported and/or have consequences been discussed?
6	Screening procedure	Were measures described that had been undertaken for standardization of screening measurements?
7	Swabbing sites	Have routine surveillance cultures included the anterior nares (or nostrils or nose) and the throat?

### Outcome definition

The infection-related outcome had to be clearly defined and described for MRSA colonization and infection [18]. Definition for denominators (e.g. patients, isolates, patient days) had to be appropriate [18]: If measurements are performed on the patient's first admission, these are expressed relative to the total number of patients; if measurements are performed for each admission, these are expressed relative to the total number of admissions. If calculations are related to isolates, it should be stated whether only the initial MRSA isolate or all subsequent isolates of a patient during the study period were included. In case of multi-site swabbing, it should be stated whether swabs were cultured separately or as a pooled sample on the same agar plate. For density rates, it should be stated whether denominators were adjusted for total patient days or number of patient-days at risk for new MRSA detection [13].

### Time unit

The outcome had to be calculated for a standardized time unit (daily, weekly, monthly, yearly) [18].

### Target population

The eligibility criteria had to be described [19].

### Participants

The number of cases potentially eligible and actually included had to be reported. A flow chart is recommended for the description of the numbers and reasons for non-participation [19].

### Observer bias

Any efforts to reduce potential sources of bias and imprecision had to be described and the consequences of any potential bias had to be discussed [18].

### Screening procedure

The sampling procedures had to be described [18] and it should be stated if swab specimens were taken by specially trained healthcare workers [20]. Detailed instructions on swab sampling should be given, i.e. use separate swabs for each sampling site of the nares [5], roll the swab three times around in the outer section of each nostril and take the swab from the posterior wall of the throat and not from the mouth [20]. Studies in which the sampling procedure was described in more detail and/or performed by a staff member after appropriate training were therefore scored with one point.

### Sampling sites

The preferred screening method in MRSA prevalence studies is to use swabs. The anterior nares are considered to be the primary colonization site for *S. aureus *[1,21]. Screening of the throat in addition to nasal swabs has increased the sensitivity of detection of *S. aureus *among carriers by 20% to 26% [20,22]. Although no standards exist for the choice of anatomical sites [4], one scoring point was given when sampling sites included at least the anterior nares and the throat, according to the recommendations by Albrich & Harbarth [5] and various national guidelines [12,23,24]. Studies relying on clinical cultures in addition to screening cultures were only awarded a point when screening cultures included samples from both the anterior nares and the throat. Surveillance studies relying exclusively on clinical cultures (e.g. blood culture of infected patients) were scored with zero points, because clinical cultures taken for therapeutic reasons have shown low sensitivity for detecting the MRSA reservoir [25].

Microbiological media for MRSA screening or use of the polymerase chain reaction were not assessed by the quality score, as no standards exist that define the most effective microbiological media [4].

Extracted data on study design, screening policy and primary MRSA outcomes are described by a narrative approach.

## Results

Table 2 summarizes studies on the prevalence of MRSA in LTC facilities. In 11 studies, surveillance data came from the same setting, nursing homes for the elderly. Two studies (No.8,11/1) included other sectors (rehabilitation center and hospital). The study population was recruited from the residents of the corresponding facility. Study duration varied between 1 and 12 months. In all 11 studies, MRSA prevalence was derived solely from surveillance cultures. Nasal swabs were performed in all studies, though only in six studies by swabbing the anterior nares. In nine studies, screening was performed as multi-site swabbing, most often by additional swabs taken from skin lesions or wounds. Swabbing of the throat was performed routinely in five studies. The screening was non-selective in all studies with regard to study population. The screening procedure was described in more detail in five studies (on a single day or on a two days period in each unit). The quality level was assigned as 'good' in two studies, as 'fair' in six studies, and as 'poor' in three studies. Prevalence in LTC facilities varied from 1% to 23%, if the percentage of MRSA among residents is taken, and from 5% to 54%, if the percentage of MRSA among *S. aureus *isolates is taken. MRSA proportions of around 20% or higher were found in UK, Northern Ireland and Belgium.

**Table 2:** MRSA prevalence among patients and residents in long-term care facilities

**NO**.	Author (First)	Study design				Screening policy		Results			**Quality Rating**^**3**^
	Country, Year of Publication	Study period, dates (months)	Study, setting (no. of units) (n)	Basic number of cases potentially eligible (n)	Study population (no. of residents) (n)	Screening methods	Swabbing sites	Number of MRSA patients colonised (C) or infected (I) (n)	Proportion of MRSA		
									**% MRSA among patients Mean (95% CI)**^**1**^**Range**^**2**^	**% MRSA among *S. aureus *isolates Mean (95% CI)**^**1**^	
1	Baldwin [36] Northern Ireland 2009	2005-2006 (9)	NH-E (45)	1.678	1.111^4^	Non-selective on one day in each unit	Nares, urine, wounds, inv.devices	267 (C)	23.3 (18.8-27.7) Range, 0 to 73		Good 1Y; 2Y; 3Y; 4Y; 5Y; 6Y; 7N
2	Barr [38] UK 2007	2005 (2)	Care homes for the elderly (39)	1.342	715^4^	Non-selective	Nose	159 (C)	22.0 (18.0-27.0) Range, 0 to 50	54.0	Fair 1N; 2N; 3Y; 4Y; 5Y; 6Y; 7N
3	Baum, von [37] Germany 2002	1999-2000 (12)	NH-E (47)	3.864	3.236^4^	Non-selective	Nares, skin defects	36 (C)	1.1 (0.75-1.47) Range, 0 to 18.2		Fair 1N; 2Y; 3Y; 4Y; 5Y; 6N; 7N
4	Brugnaro [39] Italy 2009	2006 (1)	NH-E (2 with 15 units)	570	551^4^	Non-selective on a single day in each unit	Nares	43 (C)	7.8 (5.7-10.4) Range, 0 to 18		Fair 1N; 2Y; 3Y; 4Y; 5Y; 6N; 7N
5	Cretnik [40] Slovenia 2005	2001 (1)	NH-E (1)	127	107	Non-selective	Nares, skin lesions	10 (C)	9.3		Poor 1N; 2N; 3Y; 4Y; 5N; 6?; 7N
6	Denis [33] Belgium 2009	2005 (9)	A random sample of NH-E (60)	NA	2.953	Non-selective on the same day in each unit	Nares, throat, wounds, cath. urine	587 (C)	19.5 (16.4-21.5) Range, 2 to 42.9	39.1	Fair 1N; 2Y; 3Y; 4Y; 5Y; 6N; 7Y
7	Heuck [41] Germany 2000	NA	NH-E (31)	?	1.342^4^	Non-selective	Nares, throat, wounds	32 (C)	2.4 Range, 0 to 2.9	6.0	Poor 1N; 2N; 3Y; 4N; 5N; 6N; 7Y
8	Heudorf [42] Germany 2001	1999-2000 NA	NH-E (7); geriatric RC (1)	?	NH-E: 359 RC: 42^4^	Non-selective	Nose, throat	NH-E: 8 (C) RC: 2 (C)	NH-E: 2.2 RC: 4.8		Fair 1N; 2N; 3Y; 4Y; 5Y; 6N; 7Y
9	Hoefnagels-Schuermans [43] Belgium 2002	1997 (3)	NH-E (17)	?	2.857	Non-selective; one day sampling in each unit	Nose, perineum	141 (C)	4.9 (4.38-6.09)	19.1	Poor 1N; 2Y; 3N; 4N; 5N; 6Y; 7N
10	Neuhaus [44] Germany 2002	2000-2001 (12)	NH-E (61)	?	1.057^4^	Non-selective	Nose, throat, wounds	32 (C) 3 (I)	3.0 (2.1-4.2)	6.3 (4.3-8.8)	Fair 1Y; 2N; 3Y; 4N; 5N; 6N; 7Y
11/1	Woltering [45] Germany 2008	NA (4)	NH-E (5)	441	265^4^	Non-selective on a 2-days period in each unit	Nose, throat, wounds	4 (C)	2.3 (0.8-4.9)	5.9	Good 1N; 2Y; 3Y; 4Y; 5Y; 6N; 7Y

^1^: Pooled mean calculated as a combined prevalence rate over all units with 95% CI.

^2^: Range within the individual units.

^3^: Levels for study quality: Good (= 6 and 7 points), fair (= 3 - 5 points), poor (= 1 and 2 points). Numbers belong to the questions as illustrated in Table 1.

^4^: Participants who were able to give informed consent or informed consent were given by their relatives. Abbreviations: CI, Confidence intervals; N, no; NH-(E), Nursing home for the elderly; RC, Rehabilitation centre; Y, Yes; NA, No Answer; ?, unclear or missing information.

Table 3 summarizes studies on the prevalence of MRSA in clinical settings. A total of 20 studies were included, of which one study presented two surveillance approaches (No.19). Another study was considered, which included both clinical and LTC facilities (No.11/2). Four studies were designed as multi-center surveillance studies (No.14,20,23,29). Study setting included different forms of hospitals (university, primary or tertiary care), specialized units (intensive care (ICU), surgery, neurology, vascular, emergency, orthopedics), rehabilitation centers and one laboratory facility. Study duration varied across a range from less than 1 month to 72 months, with accumulation round 4 months. Four groups of study population were found: admissions (in general or as specified admissions to ICU, to emergency, or to geriatrics); hospitalized patients (more than 24 or 48 hours in hospital); special groups of patients (of greater age, at discharge to home care, being a trauma patient); and *S. aureus *isolates of infected patients. Identification of MRSA cases was achieved in 15 studies by swabs for surveillance purposes exclusively, in three studies additionally by clinical cultures, and in another three studies exclusively via clinical cultures obtained from infected patients for therapeutic reasons.

**Table 3:** MRSA prevalence among patients in clinical settings

**NO**.	Author, First	Study design				Screening policy		Results				Comments	**Quality rating**^**4**^
	Country, Year of Publication	Study period (no. of months)	**Study setting**^**1 **^**(no. of units) (n)**	Study population	Basic no. of cases potentially eligible/no. of participants (n)	Screening methods (Origin of specimen)	Swabbing sites	No. of MRSA patients colonised (C) or infected (I) or no. of isolates (n)	Prevalence of MRSA				
									% MRSA among patients **Mean**^**2 **^**(95% CI) Range**^**3**^	**% MRSA among S.au*reus *isolates Mean**^**2**^	Density (no. of MRSA per 1,000 patients days		
12	Chaberny [46] Germany 2008	2005 (<1)	ICU (1), surgery (1), neurology (1), internal medicine (1)	Patients	700/509	On a given study day	Nares, throat, skin lesions	27 (C)	5.3 (3.5-7.7) ICU: 11.5^a ^Surgery: 5.5^a ^Neuro. 11.8 Int. med.: 5.1	19.0		^a ^Units with established admission screening	Good 1Y; 2Y; 3Y; 4Y; 5Y; 6Y; 7Y
13	Chaberny [47] Germany 2005	2002 (12)	ICU (4)	Admissions	?/188.615	On admission of patients at risk for MRSA Clinical samples^5^	n.r.	505 (C) 404 (I)	0.48		0.64^a^ 0.29^a,b^	^a ^Admissions ^b ^Noso-comial	Fair 1Y; 2Y; 3N; 4Y; 5N; 6Y; 7?
14	Chaberny [7] Germany 2007	2004 (12)	Hospital (31)	Hospita-lized patients	?/660.042	During hospital stay Clinical samples^5^	Nares	2.786 (C) 1.429 (I)			0.71^a^ 0.27^a,b^	^a ^Admissions ^b ^Noso-comial	Fair 1Y; 2Y; 3Y; 4N; 5Y; 6N; 7N
15	Eveillard [48] France 2002	2000 (1)	Acute geriatric ward (2)	Patients	244/239	On the first day of admission	Nares, wounds	35 (C)	14.6 (10.1-19.1)				Fair 1N; 2Y; 3Y; 4Y; 5N; 6N; 7N
16	Anonymous [49] Germany 2010	2008 (1)	RC (6), GH (8)	Patients	?/6.985	On admission during initial exa-mination	Nose, throat	95 (C)	RC: 2.1 GH: 1.2	RC: 9.4 GH: 5.5			Fair 1N; 2Y; 3Y; 4N; 5N; 6?; 7Y
17	Hassan [50] Ireland 2008	2005 (3)	Orthopedic ward (2)	Patients	690/686	Within 24 hrs of admission	Nose, perineum, surgical wounds	27 (C)	3.9				Fair 1N; 2N; 3Y; 4Y; 5N; 6Y; 7N
18	Hori [14] UK 2002	2000 (4)	UH (1)	Patients older than 64 years	431/342	On the 21^st ^day after admission	Nares	54 (C)	15.8				Fair 1N; 2Y; 3Y; 4Y; 5Y; 6N; 7N
19/1	Kappstein [51] Germany 2009	2000-2005 (72)	UH (1)	Patients	?	On admission Clinical samples^5^	Nose	489 (C) 38 (I)			0.42		Poor 1Y; 2N; 3N; 4N; 5N; 6N; 7N
19/2	Kappstein [51] Germany 2009	2002-2005 (42)	UH (1)	Patients	141.249/29.692	Within 48 hrs of admission	Nose, wounds	231 (C)	0.78				
20	Kresken [26] Germany 2009	2007 (1)	LF (26)	*S. aureus *isolates	?/872	Clinical samples^5^		159 isolates		20.3			Poor 1N; 2Y; 3Y; 4N; 5N; 6N; 7N
21	Lucet [52] France 2009	2003-2004 (14)	Hospitals for primary and tertiary care (16)	Patients at discharge to home care, > 48 hrs in hospital^a^	2.025/1.501	Within 3 days before discharge	Nose, chronic skin lesions	191 (C)	12.7 (11.0-14.5)			^a ^Obstetric patients excluded	Fair 1Y; 2N; 3Y; 4Y; 5Y; 6Y; 7N
22	Lucet [53] France 2005	2002 (3,5)	Acute care ward in a hospital (1)	Patients older than 75 years, > 24 hrs in hospital	1.434/797	Within 48 hrs of admission	Nose, skin breaks	63 (C)	7.9^a^ (6.0-9.8)	29.7^a^		^a ^Admissions	Fair 1Y; 2N; 3Y; 4Y; 5Y; 6Y; 7N
23	Meyer [54] Germany 2006	2001-2004 (48)	ICU (40)	S. aureus isolates of patients with noso-comial infections	?/12.238	Clinical samples^5^		2.631 isolates		21.5	4.4		Good 1Y; 2Y; 3Y; 4Y; Y5; 6N; 7N
24	Morange-Saussier [55] France 2006	2004 (4)	Vascular surgery (1)	Patients, > 24 hrs in hospital	?/308	On admission and 1 wk thereafter	Nares	13 (C)	4.2	27.0			Fair 1Y; 2Y; 3Y; 4Y; 5N; 6N; 7N
25	Gopal Rao [29] UK 2007	2004- 2005 (12)	GH (1); emergency department	Adult emergency admissions	13.826/7.801	Prior to admission	Nose, axillae	670 (C) 433 (C)	8.6^a^ 6.7^b^			^a ^Admissions ^b ^Patients	Fair 1N; 2Y; 3Y; 4Y; 5Y; 6N; 7N
26	Reilly [56] UK 2010	2008 (5)	GH (6)	Emergency (68%) and elective (32%) admissions	29.690/26.160	On admission or at pre-admission (7.5%)	Nose, wounds, invasive device sites	988 (C+I)	3.8^a^ (3.5-4.0)			^a ^Admissions	Fair 1N; 2N; 3Y; 4Y; 5Y; 6Y; 7N
27	Tai [57] UK 2004	2000 (12)	Orthopedic and trauma surgery (1)	Patients at high risk for MRSA	1.879/121	On admission Clinical samples^5^	Nose, throat, axillae, groins, wounds	10 (C) 21 (I)	1.6				Poor 1Y; 2N; 3Y; 4N; 5N; 6N; 7N
28	Thompson [58] UK 2004	2001-2004 (30)	ICU (1)	Admissions to ICU	1.472/1.361	On admission Weekly screening cycle Clinical samples^5^	Nose, groins	119 (C+I) 68 (C+I)^b^	8.7^a ^(6.1-10.2)			^a ^Admissions ^b ^Nosocomial Incidence: 1^st ^wk: 7.5%; 2^nd ^to 4^th ^wk: 20.3%	Fair 1Y; 2?; 3Y; 4?; 5Y; 6N; 7N
29	Tiemersma [3] Germany 2004	1999-2002 (36)	Hospitals (25)	*S. aureus *blood isolates	?/3.757	Blood cultures^5^		600 isolates		13.8			Poor 1Y; 2?; 3N; 4Y; 5N; 6N; 7N
30	Vos [59] NL 2009	2000-2004 (60)	UH (1)	Admissions at high risk for MRSA	?/ 21.598	On admission	Nose, throat, perineum, invasive devices, wounds	123 (C)	0.10		0.0028^a^	^a ^Related to bacteremia cases	Fair 1N; 2Y; 3Y; 4N; 5Y; 6N; 7Y
31	Walley [60] UK 2009	2003 (3)	Trauma and ortho-pedic ward (1)	Elective and trauma patients, > 48 hrs in hospital	559/323	Within 24-48 hrs of admission	Nose, perineum	78 (C)	24.0				Fair 1Y; 2N; 3Y; 4Y; 5Y; 6?; 7N
11/2	Woltering [45] Germany 2008		GH (5), RC (3)	Patients	1.321/818	On a 2-days screening period in each unit	Nose, throat, wounds	GH: 17 (C) RC: 6 (C)	GH: 3.4 (2.1-5.6) RC: 1.2 (0.4-3.3)	GH: 11.6 RC: 5.6			Good 1N; 2Y; 3Y; 4Y; 5Y; 6N; 7Y

^1^: UH = University hospital, ICU = Intensive care unit, TH = Teaching hospital, GH = General hospital, RC = Rehabilitation centre, LF = Laboratory facility, n.r. = not reported.

^2^: Pooled mean calculated as a combined prevalence rate over all units with 95% CI.

^3^: Range within the individual units.

^4^: Levels for study quality: Good (= 6 and 7 points), fair (= 3 - 5 points), poor (= 1 and 2 points). Numbers belong to the questions as illustrated in Table 1.

^5^: Specimens (blood, sputum, others) taken for diagnostic purposes. Abbreviations: CI, Confidence intervals; hrs, hours; N, no; wk, week; Y, Yes; ?, unclear or missing information.

Screening was performed at different time points: on admission within 24 to 48 hours or during hospital stay (one week after admission, on the 21^st ^day of admission, within three days before discharge to home care, or by a weekly screening cycle). Nasal swabbing was performed in 18 studies, but anterior nares were used in five studies only. Wounds were the second most frequently swabbed screening site (11 studies). Additional anatomical sites were either the throat, groin, axillae, or perineum. Considering the seven quality criteria, the quality level was assigned as 'good' in two studies (plus one already counted in Table 2; No.11), as 'fair' in 14 studies, and as 'poor' in four studies.

The prevalence rates in clinical settings varied from 0.1% to 24.0% if the percentage of MRSA among patients is taken and from 5.5% to 29.7% if the percentage of MRSA among *S. aureus *isolates is taken. In surveillance studies analyzing solely clinical samples of infected patients, the percentage of MRSA among *S. aureus *isolates varied between 13.8% and 21.5% (No.20,23,29). Prevalence density was calculated in four studies and varied between 0.4 and 0.7 MRSA-positive cases per 1,000 patient days, when all patients gave rise to the denominator (No.13,14,19), and was 4 MRSA isolates per 1,000 patient days when calculated on the basis of clinical samples of infected patients (No.23).

With regard to screening procedures, the MRSA percentage varied from less than 1% to 24% when swabs were taken within 24 to 48 hours of admission, from 3.4% to 15.8% when swabs were taken during the hospital stay, and from less than 1% to 7.9% when swabs were taken on admission of patients with a high risk for MRSA carrier status. With regard to comparable clinical settings, the MRSA percentage among patients varied from 0.48% to 11.5% in intensive care units (n = 3), from 7.9% to 14.6% in acute (geriatric) care wards (n = 2), from 1.6% to 24.6% in surgeries (orthopedic, trauma, or vascular) (n = 5), from less than 1% to 15.8% for different wards in hospitals in general (n = 1 0), and from 1.2% to 2.1% in rehabilitation centers (n = 2).

## Discussion

In this review, 31 studies on MRSA prevalence rates in endemic situations in different healthcare settings in eight European countries were analyzed. Prevalence rates, defined as the proportion of MRSA-positive patients, varied widely over a range between less than 1% and 24%. The variations in MRSA proportion were less marked between acute-care and long-term care setting, but rather between single wards in each setting. Study quality was assessed as good in only four studies. Most studies (n = 20) were assessed as being of fair quality and seven as of poor quality. The aim of this review was to present prevalence rates for endemic MRSA in all kinds of healthcare sectors, but was not fully achieved. In fact, MRSA rates are presented for a few different clinical sectors and not for a variety of LTC sectors, as in the LTC setting, surveillance was performed only in nursing homes for the elderly.

In the last 10 years, significant increases in MRSA (expressed as percentage of *S. aureus *blood isolates) have been shown in European countries [3]. Time trends for resistance proportions for MRSA have been presented by two other surveillance systems, with an increase of MRSA from 1% in 1990 to 20% in 2007 [26], and stable proportions between 20% and 26% in the years between 2001 and 2007 [27]. The MRSA rates of the studies analyzed in this review show a different distribution, with the most frequent rates being less than 10% (expressed as MRSA among patients). The range of MRSA rates may reflect differences in specific national characteristics (countries with known higher MRSA rates), but may also be caused by methodological weaknesses, as seen by the quality of the included studies. Interpretation of the results is most hampered by the heterogeneity in the study population, screening policy, study period, and calculation of the outcome.

### Study population

As study participants were not homogenous, but were members of different populations (admissions, patients, residents, and *S. aureus *isolates), it was difficult to compare MRSA rates. In the clinical setting, inclusion criteria were time-related (on admission or during hospital stay) and risk-related (patients with a high risk for MRSA carrier status). Regardless of the different inclusion criteria, all studies are prone to underestimate the MRSA reservoir. Admission-only screening does not assess healthcare-associated transmission [13], but rather provides data on imported MRSA cases [28], although transmission of MRSA in most cases happened during a previous hospital stay. Selective screening of high-risk patients on admission to the hospital or to special wards is a strategy recommended by national guidelines, but risk factors for MRSA carriage are not standardized, nor were they described by the studies. In addition, poor compliance with selective risk factor based screening has been reported [29], so that not all colonized patients may have been detected. In the case of screening programs including only groups of high-risk patients after admission to hospital, MRSA rates are not representative for the hospitalized population as a whole [13,14]. Underestimation of the MRSA reservoir is also expected from studies relying exclusively on clinical cultures taken for therapeutic reasons, as they may fail to identify 85% of the MRSA-colonized patients [25].

In the LTC setting, even if all study participants were recruited as residents of nursing homes for the elderly, comparison of the MRSA rates is difficult, because potentially eligible cases were not described by inclusion criteria. The findings of this review suggest that screening programs in LTC facilities are not assigned to admission of new residents. However, time-points for screening were used in some studies in an almost standardized way (No.1,4,6,9,11), so that these rates may present representative rates for the population of residents in the nursing home as a whole. No statement is possible for endemic MRSA in LTC settings such as home care, day-care institutions for (multiply) handicapped people, or institutions for patients with long-term artificial respiration. Only one study (No.21) investigated the MRSA clearance on discharge from hospital of home-care patients colonized with MRSA.

### Screening policy

Swabs were the preferred screening method in the included studies, but heterogeneity was found for the anatomical sites which had been swabbed. Even though the contribution of variations in sampling procedures is not clear [30], lack of sensitivity of the swabbing sites has to be considered when surveillance data from different facilities are compared [31]. For the studies using only nasal swabs, underreporting of MRSA cases is suggested, given that cultures of the nares identify only 60%-73% of the *S. aureus *carriers [20,22,31,32]. That is why in national guidelines screening of additional sites is recommended, though no consensus has been reached [4,31]. For reasons of accessibility, compliance and consistency with other investigations, it is recommended by Hori [14] that the investigation of MRSA prevalence should be confined to nasal swabs. Though this recommendation might be considered excessively strict, it is nevertheless reasonable, as it permits comparison between different studies.

The swabbing procedure is described in more detail in only a very few studies. Therefore, difference in swabbing procedures might also be a reason for the different MRSA rates. For standardization of MRSA measurement by surveillance cultures, the sampling procedures have to be considered [4] and it is recommended that the swab specimens are obtained only by specially trained healthcare workers [20].

### Study period

The range in study period, between less than one months and six years, is due to the two most often used screening modes, point prevalence and prospective screening over different study phases. As the data were generally related to total study period, results are not comparable. According to the ORION statement, infection-related outcomes should be related to regular time units rather than presented as totals for study phase [18]. There are no standards for the most effective screening mode for MRSA prevalence. Even if point-prevalence rates offer the best choice for comparison, the limitations of this method should be kept in mind, as with point-prevalence only a short-time cut-out is considered and prevalence on another day might differ [33]. Repeated point-prevalence measurements are therefore recommended in order to achieve a more comprehensive view of the endemic situation [34].

### Calculation of the outcome

Comparison of MRSA rates was hampered, as two different prevalence numerators were used, patients colonized with MRSA and methicillin-resistant isolates. A further difficulty was that the method of counting differed with regard to repeated admission or repeated MRSA-findings of a patient during the study period, which complicates the comparison.

Comparison of MRSA rates was also hampered, by the fact that the outcomes were reported either in the form of proportions or as prevalence density. For resistance proportions (expressed as MRSA among *S. aureus *isolates), consistency was observed for the clinical setting (between 13% and 20%), but these data are not available for the LTC setting. From a public health perspective, resistance proportions do not allow an unbiased estimate of the MRSA burden in the respective setting [35], and therefore density rates are recommended as a more appropriate measure for the average MRSA burden [7,27]. MRSA rates in the form of density data were presented by only a few studies. As most studies misleadingly reported incidence density using total patient days as denominators (instead of patient days at risk for new MRSA detection), the true incidence of MRSA acquisition is probably underestimated [13]. Concerning the differences in calculations of MRSA outcomes, the findings of our review are in line with the assessment of other authors, that up to now no surveillance method allowing calculation of the rate of MRSA colonization and infection has been gained acceptance as a valid method for comparisons between institutions [7].

One of the limitations in our review is that a high proportion of the retrieved articles was excluded from the review, as MRSA prevalence was not the primary objective. Inflation of MRSA hits is probably caused by a trend to add MRSA to both keyword lists and abstracts of every study of even peripheral relevance to the surveillance of antimicrobial resistance. Another limitation in our review is that the quality of the studies was not assessed by a validated score. However, the majority of the selected quality criteria have already been used to critically appraise research articles which estimate the prevalence of a disease [15].

## Conclusions

Since the recognition of MRSA as a hospital problem largely depends on swabs or clinical samples taken on admission or during hospital-stay of high-risk patients, the true case load of a hospital remains largely unknown. In order to enable comparison between different studies and different settings, MRSA rates should be assessed in a standardized way, e.g. the anterior nares should always be included and sampling should be performed by a trained person.

Accurate incidence measures using denominator data adjusted for the at-risk population are warranted for comparing MRSA studies. However, these incidence measures are difficult to obtain, especially in LTC settings. Therefore less demanding estimates of MRSA rates should be used in a standardized way in clinical and LTC settings in order to allow comparison. Consensus should be achieved, in order to define standardized procedures for MRSA surveillance in different healthcare settings.

## Competing interests

The authors declare that they have no competing interests.

## Authors' contributions

MD performed the literature search, performed the extraction of the data, and drafted the initial manuscript; FH helped with data extraction and helped with data interpretation; CP and AS performed the quality scoring and helped with data interpretation; AN critically revised the manuscript for important content. All authors read and approved the final manuscript.

## Funding

The study was funded by the Institution for Statutory Accident Insurance and Prevention in the Health and Welfare Services (BGW).

## Pre-publication history

The pre-publication history for this paper can be accessed here:

http://www.biomedcentral.com/1471-2334/11/138/prepub
